# Advancements in Genetic Biomarkers and Exogenous Antioxidant Supplementation for Safeguarding Mammalian Cells against Heat-Induced Oxidative Stress and Apoptosis

**DOI:** 10.3390/antiox13030258

**Published:** 2024-02-20

**Authors:** Muhammad Zahoor Khan, Adnan Khan, Wenting Chen, Wenqiong Chai, Changfa Wang

**Affiliations:** 1 Liaocheng Research Institute of Donkey High-Efficiency Breeding and Ecological Feeding, Liaocheng University, Liaocheng 522000, China; 2 Genome Analysis Laboratory of the Ministry of Agriculture, Agricultural Genomics Institute at Shenzhen, Chinese Academy of Agricultural Sciences, Shenzhen 511464, China

**Keywords:** heat stress, oxidative stress, apoptosis, antioxidants, mammalian reproductive cells, fertility

## Abstract

Heat stress represents a pervasive global concern with far-reaching implications for the reproductive efficiency of both animal and human populations. An extensive body of published research on heat stress effects utilizes controlled experimental environments to expose cells and tissues to heat stress and its disruptive influence on the physiological aspects of reproductive phenotypic traits, encompassing parameters such as sperm quality, sperm motility, viability, and overall competence. Beyond these immediate effects, heat stress has been linked to embryo losses, compromised oocyte development, and even infertility across diverse species. One of the primary mechanisms underlying these adverse reproductive outcomes is the elevation of reactive oxygen species (ROS) levels precipitating oxidative stress and apoptosis within mammalian reproductive cells. Oxidative stress and apoptosis are recognized as pivotal biological factors through which heat stress exerts its disruptive impact on both male and female reproductive cells. In a concerted effort to mitigate the detrimental consequences of heat stress, supplementation with antioxidants, both in natural and synthetic forms, has been explored as a potential intervention strategy. Furthermore, reproductive cells possess inherent self-protective mechanisms that come into play during episodes of heat stress, aiding in their survival. This comprehensive review delves into the multifaceted effects of heat stress on reproductive phenotypic traits and elucidates the intricate molecular mechanisms underpinning oxidative stress and apoptosis in reproductive cells, which compromise their normal function. Additionally, we provide a succinct overview of potential antioxidant interventions and highlight the genetic biomarkers within reproductive cells that possess self-protective capabilities, collectively offering promising avenues for ameliorating the negative impact of heat stress by restraining apoptosis and oxidative stress.

## 1. Introduction

Heat stress refers to physiological responses occurring due to prolonged exposures to high temperatures, often combined with elevated humidity, that cause body heat gain to exceed one’s ability to dissipate the heat [1]. It arises when the body is unable to thermoregulate properly, leading to an abnormal rise in core body temperature [2]. The fundamental origin of escalating heat stress is global warming stemming from anthropogenic climate change—the accumulation of greenhouse gases like carbon dioxide, methane, and nitrous oxides due to human activities including burning fossil fuels, deforestation, and agriculture [3]. One key mechanism is through the impairment of evaporative cooling from sweating and respiration [4]. High humidity in particular hampers heat loss via sweating and skin evaporation by reducing the vapor pressure gradient from the skin to the environment [5]. Studies show that humid heatwaves have become more frequent worldwide [6], causing instances of near-fatal and fatal hyperthermia and heat stroke by blocking the primary heat loss avenue in humans [7]. The multifaceted effects of escalating heat stress can be detrimental with widespread repercussions for health, economies, ecosystems, and more. Some major anticipated impacts include dramatic rises in heat-related compromised reproductive efficiency [8]. The deleterious effects of heat stress (HS) on reproductive function encompass a comprehensive spectrum of repercussions affecting both male and female reproductive components, spanning from the intricate mechanisms of fertilization to the critical stages of early and late embryo–fetal development. Consistently, extensive investigations have collectively unveiled the multifaceted impacts of HS on the reproductive landscape [9,10,11,12,13].

Within the sphere of male reproductive physiology, HS emerges as a pivotal factor intricately linked to male infertility, exerting a discernible influence on testicular functionality [13,14]. Notably, empirical evidence drawn from diverse studies underscores the detrimental ramifications of HS on male fertility and semen quality [15,16,17,18,19,20,21,22]. Furthermore, HS casts a shadow over the ensuing fertilization processes, as elucidated by recent investigations [23,24,25]. Recent scientific inquiries have accentuated the detrimental influence of HS on sperm quality across a spectrum of mammalian species, encompassing dogs [17], bulls [20], buffaloes [26], stallions [27,28,29], rabbits [30], pigs [31], goats [32], and human males [33,34]. Remarkably, a recurring theme in these studies underscores the adverse impact of HS-induced oxidative stress and apoptosis on the viability of Sertoli cells, spermatogonial stem cells, Leyding cells, spermatogenesis, and sperm quality [15,35,36,37,38,39,40,41,42]. Furthermore, recent publications have elucidated a compelling association between sperm quality and environmental temperature in men, underscoring the pervasive implications of environmental factors on male reproductive health [43,44,45,46]. These findings collectively reinforce the imperative need for comprehensive research into mitigating the detrimental effects of HS on reproductive function across species, as well as the exploration of potential interventions to safeguard male fertility and sperm quality under challenging environmental conditions.

**Figure 1:**
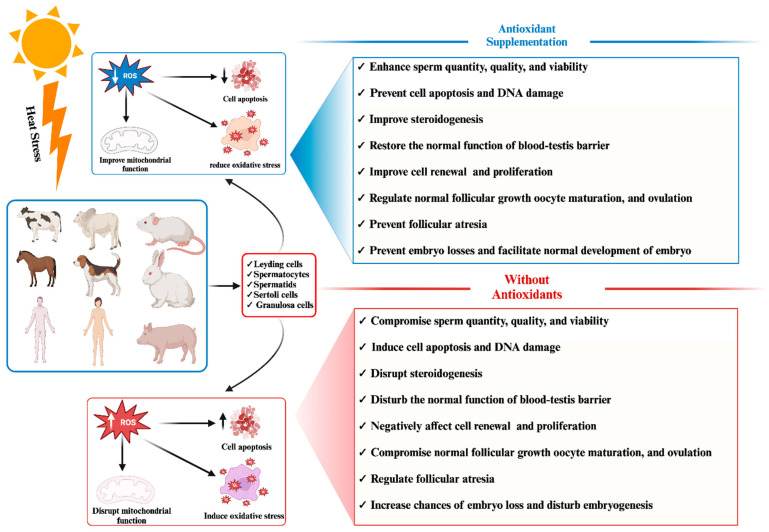
Effect of antioxidant supplementation on mammalian reproductive cells under heat stress: pre- and post-supplementation comparison.

In the United States, cattle reproduction and milk production rates experience a pronounced decline during the hot season due to HS, resulting in approximately USD 900 million in annual losses within the dairy industry [47,48]. Besides the metabolic stress associated with high milk production in dairy cows, seasonal impacts on fertility have been extensively documented [49,50]. Notably, a substantial reduction in cattle fertility during the summer months has been attributed to the thermoregulatory challenges posed by HS, leading to elevated body temperatures [51,52,53]. Furthermore, HS exerts detrimental effects on follicle quality and hormonal equilibrium, contributing to a decline in estrus [54,55,56,57,58]. Specifically, granulosa cells responsible for estradiol production are adversely affected by HS, resulting in decreased estradiol production and subsequent disruption of the estrous cycle in cattle [59,60,61]. HS has also been shown to influence corticoid levels, luteinizing hormone (LH), and plasma progesterone in cattle, with alterations in hypothalamus and pituitary gland function leading to changes in the secretion of reproductive hormones [9,62,63,64,65,66]. Moreover, HS negatively impacts oocyte quality due to its effects on the hypothalamus and pituitary gland, specifically the LH [67,68,69]. Additionally, Khan et al. [47] reported that HS significantly compromises granulosa cells, leading to suboptimal oocyte development. This is further supported by studies that found HS compromises the mRNA expression levels of Moloney sarcoma oncogene (MOS), growth factor 9 (GDF9), and POU domain, class 5, transcription factor 1 (POUF51), which are crucial for oocyte development and competence, ultimately resulting in poor-quality oocytes and impaired embryonic development [60,61,70,71,72]. Figure 1 demonstrates the adverse impact of heat stress on phenotypic traits related to mammalian reproductive traits.

In response to these challenges, numerous researchers have advocated for exogenous supplementation with antioxidants to mitigate the elevated levels of reactive oxygen species (ROS) induced by HS and enhance the antioxidant capacity of mammalian reproductive cells [30,31,47,73,74,75,76]. Consistently, positive outcomes have been observed for supplementation with antioxidants, whether in the form of herbal medicines or feed additives, in improving the efficiency of mammalian reproductive cells such as granulosa cells, Leydig cells, and Sertoli cells by alleviating oxidative stress [77,78,79,80,81,82,83,84,85]. The beneficial impact of antioxidant supplementation in mitigating the adverse effects of heat stress on phenotypic traits related to mammalian reproduction has been succinctly illustrated in Figure 1. Thus, our study aims to comprehensively investigate heat-stress-induced oxidative stress and apoptosis in mammalian reproductive cells, elucidating their manifestations. Additionally, we delve into the genetic resistance mechanisms employed by these cells to counteract heat stress, elucidating the activation of adaptive pathways aimed at ameliorating oxidative stress and preventing apoptosis. In accordance with published data, we elucidated the progression and comprehension of critical genetic biomarkers linked to the mitigation of oxidative stress and apoptosis triggered by heat stress in mammalian reproductive cells. Finally, we explore potential remedial approaches, such as exogenous antioxidant supplementation through feed additives and herbal medicines, as strategies to mitigate the detrimental effects of heat-stress-induced oxidative stress and apoptosis.

## 2. Literature Search and Selection Criteria

In the course of preparing this review article, our approach to sourcing and selecting literature was meticulously designed to meet stringent criteria. Our primary objective was to incorporate the most pertinent and up-to-date scholarly contributions, while also preserving the essential context for a comprehensive understanding. To assess the impact of heat stress on mammalian reproductive cells, we primarily focused on articles published within the last three years. However, for the exploration of genetic markers and potential antioxidants that may mitigate the detrimental effects of heat stress on reproductive cells, we extended our search to articles published between 2013 and 2023. In cases where additional supportive information was required, we even consulted articles dating back to 2000. Our selection of keywords for our search strategy was carefully thought out, encompassing the multifaceted aspects of the subject matter. These keywords included “heat stress”, “mammalian reproductive cells”, “apoptosis”, “genetic biomarkers”, “ROS” (reactive oxygen species), and “antioxidant”.

To maintain a rigorous standard, we deliberately excluded articles published in non-SCI (Science Citation Index) journals and those not written in the English language. This deliberate choice was made to ensure that the articles included in our review underwent a thorough peer-review process and were accessible to a broad academic audience. Additionally, it is important to note that we excluded book chapters and unpublished data from our discussion. Nevertheless, we incorporated fundamental insights from previously published review articles that specifically addressed topics related to the impact of heat-stress-induced oxidative stress and apoptosis on mammalian reproductive cells. 

## 3. Impact of Heat-Stress-Induced Oxidative Stress and Apoptosis on Mammalian Reproductive Cell Functionality

It is well established that heat stress induces ROS production primarily through mitochondrial dysfunction, where the electron transport chain is compromised, leading to electron leakage and the formation of superoxide radicals (O^−2^) [86,87]. Additionally, another crucial source of ROS induction during heat stress is the activation of NADPH oxidases (NOXs), particularly NOX2 and NOX4. These enzymes are responsible for generating superoxide ions in response to various stressors, including heat. The activation of NOX enzymes can occur through heat-induced signaling pathways, such as the activation of protein kinase C (PKC) and mitogen-activated protein kinases (MAPKs). The NADPH oxidases also contribute to the production of superoxide radicals during heat stress [88]. Moreover, these superoxide radicals can be further converted into other types of ROS, such as hydrogen peroxide (H_2_O_2_) and hydroxyl radicals (OH), through various reactions, including those catalyzed by superoxide dismutase (SOD) [89,90]. Consequently, heat-stress-induced ROS can also trigger lipid peroxidation, leading to the formation of lipid peroxidation products such as malondialdehyde (MDA) and 4-hydroxynonenal (4-HNE). These products are known to be cytotoxic and can disrupt cellular membranes [90,91]. These molecules, while playing roles in signaling under normal physiological conditions, can cause significant damage to cellular structures and DNA when present in excess due to heat stress [90,91,92].

Oxidative damage and cell apoptosis represent pivotal consequences affecting mammalian reproductive cells, primarily initiated by the excessive generation of ROS [93,94,95]. The accrual of ROS disrupts male reproductive functions and exerts detrimental effects on semen quality [96,97]. These detrimental effects manifest in two main modes: firstly, the overabundance of ROS depletes the cell’s scavenging capacity, impairs antioxidant enzymes, escalates lipid peroxidation, and triggers DNA damage, ultimately compromising the cell’s defense against oxidative harm. Secondly, the surplus ROS mediate molecular signaling in the mitochondria-dependent apoptotic pathway, encompassing events such as the opening of mitochondrial permeability transition pores, mitochondrial membrane depolarization, and the release of mitochondrial substances, including cytochrome C (cyto-c). This, in turn, culminates in caspase-3 activation and subsequent cell apoptosis. Recent research by Li H et al. [98] further reported that heat stress upregulated caspase-3 and caspase-9, leading to enhanced apoptosis in endometrial epithelial and glandular epithelial cells, along with alterations in HO-1 mRNA/protein and Keap1 mRNA/protein expression, and an elevated malondialdehyde (MDA) level in mouse uterine tissue.

In the context of male reproductive cells, recent investigations have extensively explored the impact of heat stress on testis morphology, antioxidant status, and testicular biosynthesis [34,99,100,101]. Sertoli cells, known for providing structural and nutritional support for developing germ cells, have been a focus of scrutiny, with studies comprehensively examining the repercussions of heat stress on male reproductive cells, including Sertoli cells, spermatogonial stem cells, and Leydig cells [102]. Wang C et al. [103] reported that HS induces oxidative stress and apoptosis in Sertoli cells, disrupting the normal spermatogenesis process. Furthermore, boar Sertoli cells exposed to elevated temperatures exhibited increased oxidative stress and apoptosis, an inhibited pentose phosphate pathway, and decreased ATP content. Molecular changes observed in boar Sertoli cells under heat stress involved the downregulation of the Kelch-like ECH-associated protein 1 (KEAP1)/nuclear factor erythroid 2-related factor 2 (Nrf2) signaling pathway (associated with enhanced antioxidants) and low levels of heat shock protein 90 (HSP90) due to the suppression of melatonin receptor 1B (MTNR1B), resulting in abnormal regulation of stabilizing hypoxia-inducible factor-1α (HIF-1α) [104]. Another study by Xue H et al. [105] found that HS primarily enhanced the lipid oxidation, oxidative stress, and apoptosis in Sertoli cells through the activation of arachidonate 15-lipoxygenase type B (ALOX15B) and the production of 8-hydroxyeicosatetraenoic acid (8-HETE) and 15-hydroxyeicosatetraenoic acid (15-HETE), with involvement of the P53-p38 pathway [105]. The disruption of arachidonic acid (AA) metabolism, a precursor to 20-carbon polyunsaturated fats, has been reported to be associated with poor spermatogenesis outcomes, as excessive AA levels altered cytomembrane structure and function and increased permeability and brittleness, potentially leading to mitochondrial changes, apoptosis, or necrosis. HS was found to significantly elevate AA levels, disrupting the function of tight junctions (TJs) essential for spermatogenesis development [106]. Additionally, AA increased MDA levels, activated p38 mitogen-activated protein kinases (P38 MAPKs), and reduced mitochondrial DNA (mtDNA). Furthermore, another study noted that heat stress induced oxidative stress in Sertoli cells by suppressing the level of nuclear factor erythroid 2-related factor 2 (Nrf2) [107]. In addition, the effects of oxidative stress on Leydig cells have been briefly reviewed in recent studies [102,108]. Heat stress treatment inhibited cell viability, induced apoptosis, increased the activity of caspase 3 and the pro-apoptotic protein Bax, and decreased the expression of anti-apoptotic protein B-cell leukemia/lymphoma-2 (Bcl-2), concurrently activating endoplasmic reticulum (ER) stress markers such as glucose-regulated protein 78 (GRP78) and CCAAT/enhancer-binding protein homologous protein (CHOP) [109].

**Figure 2:**
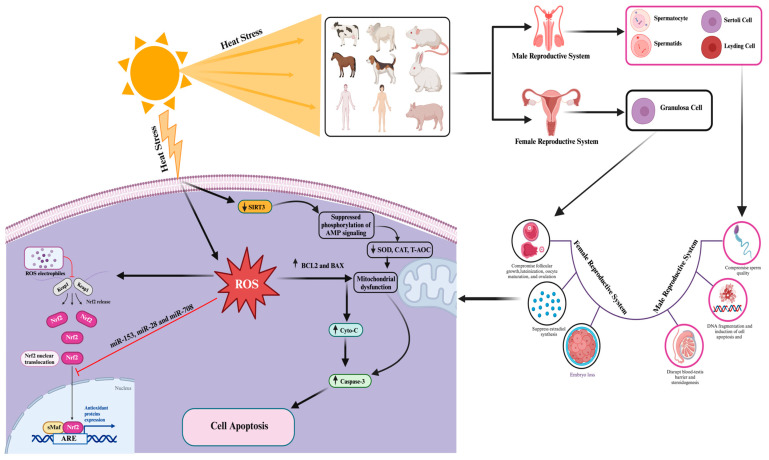
Cellular and molecular responses to heat-stress-induced oxidative stress and cell apoptosis in mammalian reproductive cells. The “→” shows direct correlation/effect, while “
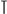
” indicates the effect has been suppressed.

The impact of heat-stress-induced oxidative stress and apoptosis on mammalian female reproductive cells has also been extensively documented in recent research [110]. In alignment with these findings, it has been observed that heat-stress-induced oxidative stress and apoptosis in bovine granulosa cells disrupt the normal secretion of estrogen, leading to disturbances in the ovarian microenvironment and subsequent interference with ovarian function [111]. Consistently, a study documented abnormal folliculogenesis including impaired ovulation, fertilization, and early embryo development [112]. Additionally, a study has reported elevated levels of ROS production in response to heat stress, resulting in increased apoptosis and even embryo death, coupled with reductions in both mitochondrial activity and membrane potential [113]. Consistently, Sammad et al. [114] have reported elevated levels of ROS and apoptosis in bovine granulosa cells under heat stress conditions. They also noted the negative regulation of several candidate genes, including heme oxygenase 1 (HMOX1), nitric oxide synthase 2 (NOS2), catalase (CAT), superoxide dismutase (SOD), B-cell lymphoma 2-like 1 (BCL2L1), glutathione peroxidase 4 (GPX4), Nrf2, aspartoacylase 3 (ASP3), peroxisome proliferator-activated receptor gamma coactivator 1-alpha (PPARGCIA), solute carrier family 16 member 3 (SLC16A3), sterol regulatory element-binding protein 1 (SERBP1), sirtuin 1 (SIRT1), AMP-activated protein kinase (AMPK), Caspase 8 (CASP8), CASP9, insulin-like growth factor 2 (IGF2), peroxisome proliferator-activated receptor alpha (PPARA), and solute carrier family 27 member 3 (SLC27A3), which are associated with apoptosis, cell proliferation, and oxidative activity of granulosa cells [114]. Furthermore, their research indicated that heat stress significantly downregulated the key anti-apoptotic and antioxidant-associated signaling pathways, including the AMP and Nrf2 signaling pathways [114]. Similarly, another study reported a significant decrease in the number of primordial follicles, an increase in the number of degenerated follicles, and a decrease in granulosa cell proliferation in response to heat stress [115]. The molecular mechanisms associated with heat-stress-induced oxidative stress and apoptosis effects on mammalian reproductive cells are summarized in Figure 2 and Table 1.

## 4. Advancement and Understanding of Genetic Biomarkers Associated with Heat Stress Resistance and Reduced Apoptosis and Oxidative Stress in Mammalian Reproductive Cells

It is well established that heat resistance in mammalian reproductive cells is mediated by a network of genes and their signaling pathways to counteract the damaging effects of heat stress. Based on published data, several genes and pathways, including HSP family genes; anti-apoptotic genes; genes encoding antioxidant enzymes; and the AMPK, ERK1/2, and Nrf2/Keap1 signaling pathways, have been found to be involved in protective mechanisms against heat-stress-induced apoptosis and oxidative stress in mammalian reproductive cells. Detailed information regarding the protective role of the aforementioned genes and pathways against heat stress in mammalian reproductive cells is provided below.

### 4.1. Role of Heat Shock Protein (HSP) Genes in Mitigating Heat-Stress-Induced Oxidative Stress and Apoptosis in Mammalian Reproductive Cells

The heat shock protein-72 (Hsp72) gene, a prominent member of the heat shock protein (HSP) family, serves as the primary inducible heat shock protein. Its baseline expression in healthy cells is minimal, but it becomes markedly upregulated in response to heat stress. Notably, HSP72 has demonstrated its ability to counteract heat-stress-induced ROS in bovine Sertoli cells when exposed to puerarin, a traditional Chinese medicinal compound [13]. In this context, HSP72 functions as both an antioxidant and an anti-apoptotic factor within Sertoli cells. It achieves this by reducing ROS production and safeguarding Sertoli cells against oxidative harm and apoptotic processes. Consistently, a study reported the protecting and self-recovering role of HSP70 in bovine oocytes after exposure to severe heat stress [119]. Furthermore, it revealed that HSP70 prevents apoptosis, supports signal transduction, increases the antioxidant protection of the embryo, as well as protecting heat-stressed maturing bovine oocytes and restoring their developmental competence. Consistently, Ho et al. [59] observed that Asparagus officinalis stem (EAS) elicited an upregulation of HSP70 and heat shock factor 1 (HSF1) expression, resulting in an augmented concentration of progesterone within heat-treated bovine cumulus–granulosa cells. Additionally, EAS demonstrated the capacity to heighten glutathione (GSH) levels, improve mitochondrial function, and mitigate ROS levels in heat-stressed bovine cumulus–granulosa cells. Notably, when HSP70 was inhibited by Ho et al., a subsequent decrease was noted in the levels of progesterone, GSH, HSF1, Nrf2, and Kelch-like ECH-associated protein 1 (Keap1). These findings collectively underscore the pivotal role of HSP70 as the principal regulator orchestrating antioxidant activity, thereby safeguarding granulosa cells against the deleterious effects of heat stress [59].

Heme oxygenase 1 (HO-1), alternatively known as heat shock protein-32 (Hsp32), is a stress-responsive enzyme with pivotal roles in maintaining iron homeostasis, fortifying antioxidant defenses, and averting apoptosis [120,121,122]. Interestingly, studies have disclosed an association between low serum levels of HO-1 and an elevated risk of polycystic ovarian syndrome [123]. Furthermore, investigations have substantiated that HO-1 attenuates heat-stress-induced apoptosis in bovine granulosa cells by curbing ROS production and activating antioxidant responses [124]. Remarkably, HO-1 modulation influences apoptotic processes, with its downregulation intensifying apoptosis and its upregulation mitigating apoptosis through the regulation of Bax/Bcl-2 expression and cleaved caspase-3 levels [111]. Additionally, HO-1 plays a cytoprotective role by influencing estrogen levels and catalyzing the breakdown of heme to generate biologically active carbon monoxide (CO). Significantly, CO elevation coincides with heightened HO-1 levels, diminished Bax/Bcl-2 ratios, and inhibition of the extracellular signal-regulated kinase 1/2 (ERK1/2) signaling pathway (Figure 2) [111]. Studies have shown that reducing antioxidant gene levels in heat-stressed (40 °C) HO-1-knockdown bovine granulosa cells leads to increased cellular apoptosis [124]. Moreover, research has elucidated that heat stress triggers the activation of Nrf2, which safeguards bovine granulosa cells from heat-stress-induced apoptosis by regulating HO-1. This, in turn, modulates ROS levels, reducing their production and subsequently suppressing oxidative stress and apoptosis [124].

### 4.2. Protective Role of SOD Genes against Heat-Stress-Induced Oxidative Stress and Apoptosis in Mammalian Reproductive Cells

**Figure 3:**
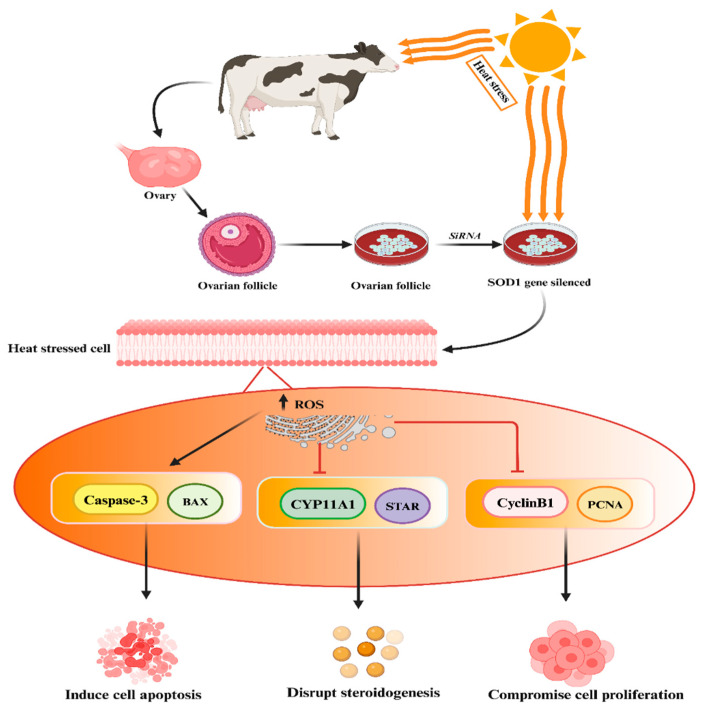
Silencing of SOD1 genes may lead to elevated levels of ROS followed by disruption of steroidogenesis, compromised cell proliferation, and increased cell apoptosis. The “→” shows direct correlation/effect, while “
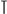
” indicates the effect has been suppressed.

The SOD genes’ protective role against heat stress has been established in mammalian reproductive cells [95,125]. Khan et al. conducted experimental studies demonstrating that the silencing of the SOD1 gene in heat-treated granulosa cells resulted in increased apoptosis, reduced cell proliferation, and decreased biosynthesis of estrogen and progesterone hormones, as depicted in the accompanying Figure 3 [95]. In addition, Faheem et al. conducted a study where they observed that under conditions of heat stress, buffalo granulosa cells demonstrated elevated expression levels of SOD2 and an enhancement in total antioxidant activity [126]. Furthermore, Faheem et al. highlighted the enhanced antioxidant capacity and cholesterol levels in granulosa cells, which likely contribute significantly to their biological function in preventing heat-stress-induced apoptosis and oxidative stress [125]. These findings suggest that SOD1 plays a key role in regulating other genes while protecting buffalo granulosa cells from the adverse effects of heat stress.

### 4.3. ERK1/2 Signaling Pathway Protects Mammalian Reproductive Cells from Heat-Stress-Induced Apoptosis

The ERK1/2 kinases are highly conserved serine–threonine kinases with widespread distribution, playing a pivotal role in cellular signaling regulation, both in normal physiological conditions and pathological states, by phosphorylating various substrates. In response to heat stress, ERK1/2 initiates a series of cascading reactions that modulate the balance between cellular survival and apoptosis molecules, thereby safeguarding a portion of male germ cells and somatic cells within the testis from destruction. Research indicates that a brief 30 min exposure to heat stress triggers an increase in phosphorylated ERK1/2 (pERK1/2) levels in immature boar Sertoli cells. This, in turn, elevates HSP70 levels and subsequently enhances the production of lactate, a primary ATP substrate crucial for the development of germ cells, by accelerating glucose metabolism [127]. Furthermore, ERK signaling exerts protective effects on pachytene spermatocytes subjected to transient heat stress by upregulating metastasis-associated 1, which counteracts the pro-apoptotic effects of p53 [128]. Conversely, inhibiting the ERK1/2 signaling pathway during heat stress significantly reduces the expression of genes such as c-fos, AP-1, and ERK2, as well as the phosphorylation of ERK1/2 and c-Fos. This inhibition is accompanied by a marked increase in c-Jun mRNA expression within Sertoli cells. Notably, the adverse effects of heat stress on the ERK1/2 signaling pathway can be ameliorated through treatment with baicalin [113]. Consistently, Wang et al. have reported that heme oxygenase 1 (HO-1) utilizes the ERK1/2 signaling pathway to suppress the expression of apoptotic genes, specifically Bax/Bcl-2, thereby restoring the normal functionality of bovine granulosa cells [111]. This interplay between ERK1/2 signaling and HO-1 underscores their pivotal roles in modulating cellular responses to heat stress, ultimately influencing cell survival and apoptosis in reproductive cells.

### 4.4. Protective Role of Nrf2 in Protection of Mammalian Cells against Heat-Stress-Induced Oxidative Stress and Apoptosis

Nrf2 is an inducible transcription factor crucial for maintaining redox signaling integrity in the face of oxidative stress [107]. Nrf2, a member of the Cap’n’Collar basic leucine zipper transcription factor family, plays a pivotal role in orchestrating antioxidant and detoxification responses through the upregulation of its downstream genes [129]. In unstressed cells, the Nrf2 is primarily located within the cellular cytoplasm and forms a complex with its inhibitory partner, Kelch-like ECH-associated protein 1 (Keap1). However, when the cellular environment encounters an elevated presence of ROS, the Keap1-Nrf2 complex undergoes dissociation, leading to the translocation of Nrf2 from the cytoplasm into the cellular nucleus [130,131]. Furthermore, the activated Nrf2 binds to the antioxidant response element (ARE) sequence, thereby stimulating the transcription of genes involved in antioxidant defenses and neutralizing ROS-induced damage [115]. Recent findings indicate that p62 can competitively interact with Keap1 at the Nrf2 binding site, altering the association, releasing ubiquitinated Nrf2, and ultimately activating the Nrf2 antioxidant systems [132,133,134].

Heat-stress-induced apoptosis triggers antioxidant responses, including autophagy and Nrf2 activation [135]. It has been observed that changes in autophagy dynamics are pivotal regulators of the Nrf2 signaling pathway’s protective role in the testis. This protection is achieved by suppressing MDA levels and promoting an antioxidant status that shields the testis from the adverse consequences of heat stress [136,137,138,139]. Notably, inhibiting Nrf2 in cells results in reduced cell viability, elevated MDA levels, and Sertoli cell death [107].

Nrf2 is known to regulate several crucial antioxidant genes, including catalase (CAT), heme oxygenase 1 (HMOX1), peroxiredoxin 1 (PRDX1), SOD1, and thioredoxin 1 (TXN1). These genes collectively enhance antioxidant activity, mitigating oxidative stress in mouse testis cells and protecting germ cells and Leydig cells from oxidative damage [56,139]. Furthermore, recent research has shown that heat-stress-induced ROS overproduction suppresses the expression of antioxidant genes (SOD, CAT, NQO1, and GSH-Px) in uterine tissue [98]. In Sertoli cells, elevated ROS levels due to heat stress increase MDA levels and decrease antioxidant enzyme levels [140]. Additionally, heat stress has been found to increase the expression of apoptotic markers such as Fas, FasL, caspase 3, and caspase 9 in mouse Sertoli cells [140]. Consequently, the Keap1/Nrf2 signaling pathway has been significantly associated with the protective effects observed in mouse uterine tissue, marked by increased levels of antioxidant genes [98].

Moreover, oxidative stress influences various important signaling pathways, including the nuclear factor erythroid 2-related factor 2 (Nrf2)/Keap1 signaling axis in the testis [141]. A recent study highlights Nrf2’s protective role in safeguarding mouse Sertoli cells from heat-induced oxidative stress through the Nrf2/Keap1 signaling pathway [107]. Similarly, another investigation revealed that Nrf2 significantly reduces caspase 3 levels, subsequently reducing cell death induced by heat stress treatment in Sertoli cells [137]. Under conditions of severe heat stress, the heightened expression of Keap1 and NFE2L2 facilitates the regulation of genes associated with antioxidants by forming complexes with the ARE, thus establishing a defensive mechanism against heat stress within bovine endometrial epithelial cells [142]. These findings collectively underscore the critical role of Nrf2 in mitigating oxidative stress and apoptosis in various cellular contexts, particularly under heat stress conditions.

### 4.5. Role of Adenosine 5′-Monophosphate-Activated Protein Kinase (AMPK) in Self-Recovery from Heat-Stress-Induced Oxidative Stress and Apoptosis

It is well established that AMPK signaling plays a key role in the tight junctions (TJs) and cell proliferation of testis Sertoli cells [143,144,145,146,147,148]. In addition, Ni et al. [147] highlighted that Sertoli cells play a key role in lactate supply, maintenance of cell junctions, and support for germ cells’ mitosis and meiosis. The AMPK signaling pathway regulates the dynamics of tight junctions and adherens junctions; the proliferation and meiosis of germ cells; and the energy metabolism, proliferation, and lactate production of Sertoli cells [149]. Once this balance is disrupted, the microenvironment of the testis and the quality of sperm will be affected. When α1AMPK was conditionally knocked out in mouse SCs, the mutant mice still showed an abnormal phenotype, including thin-head spermatozoa, reduced expression of junctional proteins (β-catenin, vimentin, occludin, and ZO-1), and deregulation of energy homeostasis [143,145,147,148]. Consistently, a study has documented that curcumin (natural antioxidant and anti-inflammatory compound) supplementation rescues porcine Sertoli cell impairment and TJs by inhibiting the NOD-like receptor family pyrin domain-containing 3 (NLRP3) inflammasome through the AMPK/SIRT3/SOD2/mtROS signaling pathway [150]. Heat stress can cause dysfunction of TJs in porcine testis reversibly via Ca^2+^/calmodulin-dependent protein kinase kinase B (CaMKKB)-induced inhibition of the AMPK signaling pathway. Consistently, Yang et al. [148] treated SCs from 3-week-old piglets at 43 °C for 0.5 h, and this hyperthermia treatment inhibited the AMPK signaling pathway, inhibiting the expression of CLDN11, JAMA, occludin, and especially ZO-1 in porcine SCs [148]. In addition, it was observed that normal Sertoli cell function was restored after 48 h due to AMP signaling [145].

## 5. Role of Exogenous Antioxidant Supplementation in Relieving Heat-Stress-Induced Oxidative Stress and Apoptosis in Mammalian Reproductive Cells

Numerous research studies have provided substantial evidence supporting the advantageous effects of supplementation with both natural and medicinal antioxidants in mitigating heat-induced apoptosis and oxidative stress within mammalian reproductive cells, as well as improving reproductive phenotypic traits (Table 2) [22,30,151,152]. Antioxidant treatment has been shown to significantly reduce oxidative stress by lowering ROS levels in oocytes, consequently enhancing embryo quantity and quality [84]. Furthermore, dietary supplementation with antioxidants has been found to improve antioxidant status by elevating the activity of SOD and CAT, leading to enhanced reproductive performance. This includes reduced MDA concentration in seminal plasma, increased total antioxidant capacity (TAC) concentration in seminal plasma, elevated total functional sperm counts, higher percentages of integrated sperm membranes, improved sperm motility, and enhanced viability [30]. Recent studies have highlighted several natural and synthetic antioxidants, including sulforaphane, periplaneta americana peptide, resveratrol, astaxanthin, growth hormones, melatonin, and celastrol, that can effectively prevent the apoptosis of granulosa cells and enhance their antioxidant activity, ensuring their normal functionality [153,154]. An overview of the impact of various antioxidant supplementation strategies on safeguarding mammalian reproductive cells from the adverse effects of heat-stress-induced oxidative stress and apoptosis is summarized in Table 2.

Baicalin, a flavonoid derived from the dried root of Scutellaria baicalensis Georgi, a traditional Chinese herbal medicine, possesses pharmacological properties, including antioxidative activity [155,156]. A study demonstrated that baicalin treatment significantly increased antioxidant enzyme activities (SOD, CAT, and GSH-Px); Nrf2 protein levels; and Nrf2, NAD(P)H quinone dehydrogenase 1 (NQO1), and glutamate-cysteine ligase catalytic subunit (GCLC) mRNA expression levels in a heat-treated group [98]. Additionally, baicalin reduced uterine epithelial cell apoptosis, MDA content, caspase-3 and caspase-9 levels, and Keap1 protein expression and enhanced HO-1 mRNA expression in heat-treated mice. This collectively suggests that acute heat stress induces oxidative damage and apoptosis in mouse uterine tissue, while baicalin protects the uterine tissue from these injuries, possibly through the Keap1/Nrf2 signaling pathway [98]. In line with the aforementioned study, another investigation reported a significant increase in antioxidant enzyme activities (SOD, CAT, and GSH-Px) and a decrease in MDA content in heat-treated mice supplemented with baicalin [140]. Moreover, it documented that heat stress induces macroscopic and apoptotic changes in testicular tissue, which are alleviated by baicalin through the enhancement of antioxidative enzyme activities. This may lead to an improved spermatogenesis process through the inhibition of the Fas/FasL pathway. Similarly, it has been reported that baicalin ameliorates heat-stress-induced cell apoptosis by modulating cell survival rates through the activation of the Fas/FasL pathway and the upregulation of Hsp72 expression in bovine Sertoli cells. In summary, protein levels of Hsp72 increased, while cell apoptotic rates and the expression of Fas, FasL, and caspases 8 and 3 decreased in Sertoli cells pretreated with various concentrations (0.1, 1, 10, 20 μg/mL) of baicalin [157]. Furthermore, another experiment found that baicalin treatment (1 and 10 μg/mL) significantly enhanced calf Sertoli cell survival rates, consequently increasing the expression of glial cell line-derived neurotrophic factor (GDNF) and stem cell factor (SCF) [157]. The blood–testis barrier (BTB) formed by Sertoli cells is a critical biological barrier that maintains spermatogenesis and provides a favorable microenvironment for this process, and heat stress has been shown to damage the integrity of this barrier [158].

Anthocyanins are a group of natural flavonoids widely distributed in various plant sources, including fruits, vegetables, grains, and other botanicals [159]. Multiple research studies have elucidated the antioxidative properties of anthocyanins and their correlation with enhancements in spermatogenesis processes [160,161,162,163,164]. Notably, cyanidin-3-O-glucoside (C3G) and protocatechuic acid (PCA) have emerged as prominent anthocyanins found in dietary sources, recognized for their robust antioxidant capabilities in supporting male reproductive health [159,162,165] and uterine epithelial cells [166]. In harmony with these findings, Cai et al. [99] reported that the administration of C3G (100 mg with heat stress) and PCA (100 mg with heat stress) effectively restored the external diameter and thickness of seminiferous tubules, alleviating atrophy and vacuolation caused by heat stress (43 °C for 30 min) in mice. Moreover, C3G and PCA displayed a capacity to enhance testicular heat stress tolerance by mitigating excessive eIF2α phosphorylation and stress granule formation. These compounds also exhibited the ability to enhance the testicular antioxidant system and regulate the IRE1α-XBP1 pathway, ultimately contributing to the amelioration of spermatogenesis dysfunction and testicular damage [99]. Consequently, anthocyanins have demonstrated the capacity to modulate ROS generation, mitigate damage to mitochondrial membrane potential, and exert an influence on testosterone levels [160]. Furthermore, among the various anthocyanin compounds evaluated, Cy-3,5-diglu with diglycoside emerged as a standout performer in terms of its antioxidative prowess. This compound exhibited notable efficacy in ameliorating cellular dysfunction and, significantly, in upregulating the expression of the steroidogenic acute regulatory protein (StAR). A direct binding interaction between anthocyanins and StAR underscores the potential mechanistic basis for the observed outcomes.

Puerarin, a bioactive isoflavone glucoside derived from radix Puerariae, a traditional Chinese herbal medicine, has garnered attention for its protective effects on reproductive cells, specifically bovine Sertoli cells, against heat-induced stress due to its antioxidative properties [13]. Furthermore, it was revealed that puerarin effectively attenuated heat-stress-induced oxidative damage and apoptosis in bovine Sertoli cells by suppressing reactive oxygen species production and upregulating the expression of Hsp72 [13]. Puerarin alleviates oxidative stress by enhancing the level of Wnt/β-catenin signaling pathway activity and SOD, CAT, Nrf2, and GPx activities and reducing the Bcl-2/Bax ratio. Furthermore, puerarin treatment significantly reduced the level of NOX4 and H_2_O_2_-derived oxidative stress by enhancing the MAPK signaling pathway.

Curcumin, a well-researched compound, is recognized for its innate antioxidant and anti-inflammatory properties [167]. Its versatility has led to investigations into its potential therapeutic applications, including those related to male reproductive health [168,169]. Notably, studies have indicated that curcumin-loaded iron particles have a substantial impact on enhancing testicular parameters, including testis volume, seminiferous tubule length, and various sperm characteristics [170]. This improvement extends to critical stereological parameters such as spermatogonia, primary spermatocytes, round spermatids, and Leydig cells, ultimately resulting in elevated serum testosterone levels. Importantly, these benefits persist even in the presence of testicular heat stress conditions, including temperatures as high as 43 °C. Furthermore, curcumin treatment has been associated with enhanced gene expression of c-kit, stimulated by retinoic acid gene 8 (STRA8), and PCNA in spermatogonia cells, further corroborating its favorable effects [170]. Recent research has also documented curcumin’s protective impact on porcine Sertoli cells under heat stress conditions [170]. Additionally, it was found that curcumin safeguards the tight junctions (TJs) of Sertoli cells by inhibiting the NLRP3 inflammasome through the AMPK/SIRT3/SOD2/mtROS signaling pathway [170]. In alignment with these findings, Pakesh et al. reported that curcumin prevented germ cell apoptosis in mouse testes by inhibiting the expression of the Bcl-2 gene while increasing the expression of Bax, miRNA-21, and circRNA0001518 [151]. Furthermore, research has indicated that curcumin positively influences sperm parameters, including sperm concentration, mass motility, sperm motility, sperm viability, cell membrane integrity, and plasma testosterone concentration. This is coupled with an improvement in the antioxidant response, marked by enhancements in total antioxidant capacity and glutathione peroxidase levels in goat males exposed to heat stress conditions [152,171]. Additionally, a study highlighted the protective effects of curcumin, administered intraperitoneally at a dosage of 50 mg/kg, in preserving reproductive cells in rats by modulating the Nrf2/ARE signaling pathway and elevating the levels of GSH-Px and SOD while suppressing the concentration of MDA [172]. Moreover, curcumin has demonstrated protective effects in testes [173] and human sperm preservation [174].

Betaine, known for its antioxidant properties [175], has shown positive effects on reproductive outcomes in various animal studies under heat stress, including in mice [176,177], sheep [178], cattle [179], and boars [180,181]. Betaine supplementation was found to be associated with improvement in the quality of epididymal spermatozoa in mice deficient in methylenetetrahydrofolate reductase (MTHFR) [182]. The metabolism of betaine through betaine homocysteine methyltransferase (BHMT) leads to the production of S-adenosyl methionine (SAM), a crucial component for creatine synthesis, essential for sperm motility and function, as well as DNA, RNA, and histone methylation [182,183]. A study conducted on rats subjected to a 42 °C treatment for 30 min, with betaine administration (250 mg/kg per day), revealed a significant upregulation of betaine-dependent metabolic pathways in the testes, including creatinine biosynthesis. This resulted in improvements in both the quantity and quality of epididymal spermatozoa and the repair of germinal epithelium [177]. The study highlighted betaine’s beneficial effects on improving epididymal spermatozoa in intact mice and its potential to mitigate heat-stress-induced complications in spermatogenesis [177]. Consistently, a recent study demonstrated that treatment with 5 mM of betaine for 24 h can prevent oxidative stress induced by high glucose levels in mouse Leydig cells [184]. Furthermore, it was found that betaine significantly upregulated the expression of key genes involved in steroidogenesis, such as 3β-HSD, StAR, P450scc, and LH receptor, which had been downregulated by heat stress treatment. Additionally, betaine downregulated the expression of ER-stress-related genes, including GRP78, CHOP, ATF6, and inositol-requiring enzyme 1 (IRE1), ultimately enhancing cell viability, attenuating endoplasmic reticulum stress, restoring testosterone production, and facilitating steroidogenesis in Leydig cells [184]. Another study observed that betaine supplementation effectively counteracted the negative effects of heat stress, including increased cell apoptosis; elevated activity of caspase-3 (an apoptosis-related modulator); reduced activity levels of antioxidant enzymes like SOD, CAT, and GSH-Px; and an increase in MDA levels [185]. Additionally, Xiong et al. reported that betaine inhibited the protein levels of critical ER stress markers such as CHOP and GRP78 in mouse Leydig cells exposed to heat stress. The treatment with betaine significantly restored diminished testosterone production in response to heat stress and increased serum testosterone concentration in mouse Leydig cells [185].

Ginseng, specifically Panax ginseng Meyer, known as Korean red ginseng (KRG), holds a prominent place in traditional herbal medicine due to its reputed ability to enhance libido and improve male fertility [96]. A noteworthy study conducted on rats revealed that the administration of extracts from KRG, particularly ginsenoside Rg3, during prolonged exposure to heat stress resulted in a significant upregulation of protein and mRNA levels of crucial antioxidant enzymes within the testes. These enzymes, such as glutathione peroxidase 4, glutathione S-transferase mu 5 and peroxiredoxin 4, were restored to levels close to normal. Furthermore, the daily administration of KRG at a dosage of 100 mg/kg effectively counteracted the adverse changes induced by heat stress on the antioxidant index in the testes. This intervention enhanced the resistance of the testes to heat-induced oxidative stress, ultimately improving testicular physiological function and, by extension, creating a more favorable environment for sperm production [186]. Consequently, KRG emerges as a promising therapeutic agent for addressing male infertility associated with hyperthermia. In a parallel study, heat stress was observed to reduce the expression levels of vital components related to antioxidant defense (GSTM5 and GPX4), spermatogenesis (CREB1 and INHA), and sex hormone receptors (androgen receptor, luteinizing hormone receptor, and follicle-stimulating hormone receptor). However, treatment with pectinase-treated Panax ginseng effectively mitigated these detrimental changes in the testes of mice [187]. The intragastric administration of Panax ginseng leaves at dosages of 150 and 300 mg/kg for a duration of 14 days yielded substantial protective effects against apoptosis, notably through the modulation of Bcl-2 and caspase protease family members. Additionally, it suppressed the hypoxia-inducible factor-1α (HIF-1α) and mitogen-activated protein kinase (MAPK) signaling pathways, thereby safeguarding against testicular damage caused by heat stress in mice [188]. Another study highlighted the protective potential of Ginsenoside Re (GRe), a primary bioactive component of ginseng, in preserving the in vitro maturation of porcine oocytes under heat stress conditions. The administration of Ginsenoside Re was associated with a reduction in the expression of apoptotic-associated genes and an enhancement of antioxidant activity, mediated through the regulation of Nrf2 in porcine oocytes [189,190].

Fisetin, recognized as an antioxidant compound, exhibits promise in mitigating the effects of testicular hyperthermia. A study by Pirani et al. [191] revealed that fisetin supplementation, initiated just before heat exposure and continued for 15 consecutive days afterward, led to notable improvements in various testicular parameters. These improvements included testicular volume, spermatogonia density, primary spermatocyte density, and round spermatid density, as well as the density of Sertoli and Leydig cells. Furthermore, fisetin positively influenced sperm parameters and the biochemical properties of testicular tissue. These beneficial effects were accompanied by a significant increase in the expression of the c-kit gene and a concurrent decrease in the expression levels of HSP72 and NF-kβ genes, Caspase3 protein, and DNA fragmentation index (DFI) in sperm cells [191].

Wuzi Yanzong Pills (WYPs), a traditional Chinese medicine formula, have exhibited promise in safeguarding the BTB against the detrimental effects of heat stress [192]. Treatment with WYPs notably increased the viability and proliferation of Sertoli cells, along with the proliferation marker Ki67. Additionally, WYPs promoted the maturation of Sertoli cells, as evidenced by increased androgen receptor (AR) expression and decreased cytokeratin 18 (CK-18) levels. Importantly, WYPs demonstrated their effectiveness in preserving Sertoli cell viability and proliferation while ameliorating dedifferentiation and the damage to BTB proteins, including zonula occludens 1 (ZO-1) and occludin, induced by heat stress via the Akt signaling pathway. These findings lend theoretical support to the potential role of WYPs in managing dyszoospermia and male infertility [192]. Moreover, a research investigation underscored the advantageous impacts of supplementation with red grape (Vitis vinifera) juice in alleviating oxidative stress and apoptosis induced by heat stress within the testicular tissue [193]. This supplementation enhanced the expression of key antioxidant genes, including catalase (CAT), SOD, and GPX, while suppressing the apoptotic enzyme caspase-3. Additionally, red grape juice contributed to the prevention of sperm degeneration and an improvement in sperm count [193].

The edible herb Angelica keiskei, commonly known as Ashitaba, boasts two major functional polyphenolic compounds, xanthoangelol and 4-hydroxyderricin, known for their antioxidant activity [194,195]. Correspondingly, research demonstrated that Angelica keiskei powder (at a dosage of 57.5 mg/kg) and its functional component, xanthoangelol (at a dosage of 3 mg/kg), significantly prevented heat-stress-induced impairment in sperm parameters, including the densities of motile sperm and progressive sperm (>25 μm/sec), as well as the amplitude of lateral head displacement. Furthermore, the expression of widely expressed heat shock proteins (HSPs) such as Hspa1a, Hspa2, and Hsp40, along with the antioxidant enzyme glutathione synthase, was elevated in the testes of mice, ultimately improving male fertility [194]. These findings collectively underscore the potential of various natural compounds, including ginseng, fisetin, Wuzi Yanzong Pills, red grape juice, and Angelica keiskei, in safeguarding testicular health and mitigating the adverse effects of heat stress, thereby offering promising avenues for addressing male infertility and related conditions.

Selenium, a vital element, plays a pivotal role in maintaining reproductive health by acting as a constituent of key proteins, such as glutathione peroxidase, and participating in various structural and functional processes within the testis, epididymis, and sperm [196,197,198]. Notably, glutathione peroxidases GPx1 and GPx3 are expressed in the epididymal epithelia and sperm, acting as crucial defenders against oxidative stress. They protect the epididymal parenchyma and mature sperm from oxidative damage. In contrast, GPx4 serves as a guardian for developing sperm, shielding them from DNA damage due to oxidative stress. Additionally, it contributes to the structural integrity of the middle mitochondrial sheath of sperm, a key factor for sperm stability and motility [199].

Empirical evidence suggests that dietary supplementation with 0.3 mg of organic selenium per kilogram of dry matter in the basal diet for rabbits can enhance heat tolerance and overall physiological well-being. This supplementation leads to improved semen quality and fertility outcomes [200]. Similarly, in heat-stressed roosters, incorporating organic selenium into their diets increases sperm count and vitality, reduces sperm mortality rates, and enhances the antioxidant status of seminal plasma, ultimately improving seminal fluid quality [201]. Selenium has also been shown to alleviate heat-induced apoptosis in granulosa cells in murine models [202]. Heat exposure upregulates the expression of apoptosis-related genes and markers of ER stress in granulosa cells. However, selenium treatment mitigates heat-induced apoptosis, improves estradiol levels, and acts as a guardian, protecting granulosa cells from apoptosis induced by heat stress through the inhibition of the ER stress pathway [202]. Consistent with these findings, selenium supplementation enhances the reproductive efficiency of male rabbits under natural heat stress conditions. This supplementation results in elevated blood serum concentrations of total protein, albumin, glucose, and glutathione peroxidase, along with improved semen quality characteristics and reproductive performance [200,203,204]. A recent study by El-Ratel et al. [80] highlighted the impact of selenium nanoparticle (SeNP) supplementation on various aspects of reproductive biology. SeNP supplementation leads to improved litter size, viability rates, hemoglobin levels, hormonal profiles, antioxidant capacity, and immunological parameters. It also enhances sexual receptivity, pregnancy rates, embryo quality, and overall reproductive capacity in female rabbits. SeNPs act as potent antioxidants, enhancing heat regulation and reproductive function through multiple mechanisms [80]. In summary, selenium plays a crucial role in preserving reproductive health by protecting against oxidative stress, enhancing semen quality, and improving fertility outcomes, especially under heat stress conditions. Its supplementation, either as organic selenium or selenium nanoparticles, offers promising avenues for maintaining reproductive function and overall well-being in various animal models.

Recent studies have highlighted the beneficial effects of dietary L-arginine (L-Arg) supplementation on boar semen quality and libido [205,206]. Notably, a combination of heat treatment and 2 mg kg^−1^ L-Arg treatment for 18 days significantly improved serum testosterone levels, catalase activity, total superoxide dismutase activity, and glutathione peroxidase activity. It also upregulated the expression of steroidogenesis-related genes such as steroidogenic acute regulatory protein (Star), steroidogenic factor-1 (Sf1), 17β-hydroxysteroid dehydrogenase 3 (Hsd17b3), and 17α-hydroxylase/17,20-lyase (Cyp17a1) in the testes, thereby enhancing the antioxidant system and testosterone-synthesis-related genes [205]. Similarly, intramuscular administration of 5 mg/kg L-arginine dissolved in 2 mL of normal saline significantly improved antioxidant responses and increased plasma testosterone concentrations in heat-stressed rams [207].

Alpha-lipoic acid (ALA) has emerged as a potent antioxidant that scavenges ROS and provides reduced GSH, thus inhibiting the formation of free radicals to maintain cellular redox homeostasis. Recent research has demonstrated ALA’s ability to prevent heat-induced apoptosis in porcine oocytes by regulating heat shock factors (HSFs) and mitigating oxidative and endoplasmic reticulum stresses [208]. In addition, it was found that ALA significantly upregulated the level of Bcl-2 and glutathione (GSH) and reduced the expression of caspase 3. Similar findings have been reported in cows [209,210], highlighting ALA’s potential to improve embryo quality and enhance cryotolerance by reducing ROS production in the face of heat stress. Consequently, ALA markedly enhances the protective response against heat-induced histomorphological alterations in the testes and attenuates the reduction in testosterone synthesis by augmenting the function of antioxidative enzymes (CAT, SOD, and GPx), mitigating endoplasmic reticulum stress-associated apoptotic signaling (Caspase 3, Bcl-2, and Bax), and promoting the expression of steroidogenic genes including steroidogenic acute regulatory protein (StAR) and 3β-hydroxysteroid dehydrogenase (3β-HSD) in chickens [211].

Recent research by Tripathi et al. [84] demonstrated that supplementation with antioxidants such as alpha-tocopherol, sodium selenite, melatonin, and ascorbic acid during in vitro maturation (IVM) reduced oxidative stress by decreasing ROS levels in oocytes. This supplementation improved embryo quantity and quality while also regulating the expression of stress-related genes (SOD-1 increased and MDA reduced), growth-related genes (GDF-9 and BMP-15 increased), and apoptosis-related genes (BCL-2 and BAX decreased). Moreover, the combination of dietary vitamin E and organic selenium demonstrates a synergistic effect in attenuating lipid peroxidation and enhancing the antioxidant environment in poultry seminal plasma. This results in increased sperm count and vitality and reduced sperm mortality under heat stress conditions [212]. Furthermore, vitamin C treatment has been observed to counteract the detrimental effects of heat on Sertoli cells in rats. It inhibits apoptosis, lipid peroxidation, and lactate dehydrogenase (LDH) activity, while enhancing the expression of protective factors such as CryAB, Hsp27, Hsp70, and Hsp110 [213]. Selenium (0.5 ppm selenium/kg diet) and vitamin E (200 mg alpha-tocopherol/kg diet) supplementation have also shown promise in inhibiting apoptosis, improving antioxidant status, and enhancing spermatogenesis in mice testes following scrotal hyperthermia (42 °C, 30 min), along with elevated expression of GPX [214].

Melatonin exhibits dual antioxidant mechanisms, directly scavenging ROS and activating the cellular antioxidant defense systems [215]. Studies conducted on heat-stressed mouse testes have demonstrated melatonin’s ability to mitigate heat-induced oxidative stress and preserve the structural integrity of Sertoli cell tight junctions [216]. Notably, some studies reported the concentration range of melatonin in porcine seminal plasma to be 2.75–35.61 pg/mL, while researchers have identified melatonin membrane receptors in the testes of various species, including sheep, pigs, mice, and humans, underscoring melatonin’s role in regulating spermatogenesis [215,217]. In line with these observations, a recent study by Deng C et al. [104] has elucidated that melatonin treatment effectively alleviates heat-stress-induced oxidative stress and apoptosis. This protective effect is mediated through the activation of the Kelch-like ECH-associated protein 1 (KEAP1)/NF-E2-related factor 2 signaling pathway, thereby enhancing the antioxidant capacity. Furthermore, melatonin amplifies the expression of heat-shock protein 90 (HSP90) by acting through melatonin receptor 1B (MTNR1B), resulting in the stabilization of hypoxia-inducible factor-1α (HIF-1α). This activation of the HIF-1α signaling pathway facilitates glycolysis, promotes the pentose phosphate pathway, and enhances cell viability. Importantly, melatonin has been shown to reprogram glucose metabolism in Sertoli cells through the MTNR1B–HSP90–HIF-1α axis, offering a theoretical framework for preventing heat-induced testicular injury [104]. Consistently, research has corroborated the protective function of melatonin against thermal stress [218]. It was found that melatonin upregulates the expression of antioxidant genes and suppresses apoptosis by inhibiting the expression of apoptotic genes in sheep granulosa cells. Additionally, Guo et al. [219] reported that long-term treatment with 50 mg/kg of melatonin confers protective effects against stress-induced DNA damage and apoptosis in germ cells. This treatment leads to spermatogenic cell regeneration, restoration of testicular weight, and the reestablishment of gap junctions and tight junctions after heat stress. It further promotes hollow seminiferous tubule filling through engulfment and the activation of the cell motility 1 (Elmo1)/RAC1 pathway. In a relevant context, it has been documented that heat stress at 42 °C induces testicular cell apoptosis in mice through the activation of the activated transcription factor 6 (ATF6) and protein kinase R-like endoplasmic reticulum kinase (PERK) signaling pathways [220]. Notably, melatonin administration (20 mg/kg melatonin for 7 consecutive days before heat treatment) significantly inhibits ATF6/PERK signaling, thereby preventing testicular cell apoptosis [220]. Furthermore, it has been demonstrated that a high level of ROS induces oxidative damage to human spermatozoa, leading to reduced sperm motility and viability [221]. However, pretreatment of human spermatozoa with melatonin mitigates this damage by suppressing sperm mitochondrial ROS generation, increasing mitochondrial membrane potential, reducing the formation of the lipid peroxidation product 4-HNE, and minimizing sperm DNA damage and apoptosis. In summary, the cumulative evidence suggests that melatonin holds promise as a potential therapeutic option for addressing male infertility attributed to heat-induced oxidative stress [222].

**Table 2:** Summary of studies associated with strategies for prevention of heat-stress-induced oxidative stress and apoptosis in mammalian reproductive cells.

Treatment	Biological Effect	Species	Reference
Curcumin-loaded iron particle (240 μL) + scrotal hyperthermia treatment (43 °C) for 20 days)	⟡ Significantly increased testis volume, seminiferous tubule length, sperm parameters, and stereological parameters (spermatogonia, primary spermatocytes, round spermatids, and Leydig cells), resulting in elevated serum testosterone levels. ⟡ Upregulated the expression of c-kit, STRA8, and PCNA genes, leading to enhanced antioxidative capacity within the testes.	Mouse testis	[170]
Puerarin treatment	⟡ Successfully suppressed the production of reactive oxygen species and reduced oxidative damage in bSCs exposed to heat stress. ⟡ Demonstrated enhanced activities of superoxide dismutase, catalase, and glutathione peroxidase, while concurrently inhibiting malondialdehyde content. ⟡ Effectively inhibited the initiation of the mitochondria-dependent apoptotic pathway, as evidenced by alterations in the Bax-to-Bcl-2 ratio, mitochondrial membrane potential, cytochrome C release, caspase-3 activation, and apoptotic rate. ⟡ Showed an increase in Hsp72 expression.	Bovine Sertoli cells	[13]
Heat treatment at 43 °C for 14 days was followed by oral supplementation with fisetin (10 mg/kg/day)	⟡ Fisetin treatment significantly increased testicular volume, the density of spermatogonia, primary spermatocytes, round spermatids, and Sertoli and Leydig cells, as well as sperm parameters. It also positively influenced the biochemical properties of testis tissue. ⟡ Elevated the expression of the c-kit gene. ⟡ Reduced the expression of HSP72 and NF-kβ genes, Caspase3 protein, and DNA fragmentation index (DFI) in sperm cells.	Mouse Sertoli cells	[191]
43 °C heat treatment/30 min/day for 14 days followed by *M. roxburghianus* (400 mg/kg) for 14 d	⟡ Suppressed lipid peroxidation, restored antioxidant enzyme levels and testosterone levels, promoted spermatogenesis, and increased cell proliferation activity.	Mouse Sertoli cells	[223]
Vitamin C treatment	⟡ Pretreatment with Vitamin C protected Sertoli cells against heat stress by reducing oxidative stress and inducing heat shock protein expression.	Mouse Sertoli cells	[213]
Selenium supplementation (0.3 mg OSe/kg DM diet)	⟡ Enhanced the reproductive efficiency of males, significantly increasing blood serum concentrations of total protein, albumin, glucose, and glutathione peroxidase in natural heat stress conditions in rabbits. ⟡ Selenium-fed rabbits exhibited lower reaction times, higher total functional sperm counts, and higher percentages of integrated sperm membranes.	Rabbit Sertoli cells	[200]
Baicalin treatment	⟡ Ameliorated cell apoptosis induced by heat stress through modulation of the cell survival rate via the Fas/FasL pathway. ⟡ Activation and upregulation of Hsp72 expression in bSCs.	BSCs	[157]
Baicalin treatment	⟡ Protection of testicular tissue from damage caused by heat stress. ⟡ Promotion of the process of spermatogenesis, which was impaired by heat stress. ⟡ Enhanced the antioxidant response of the testis by elevating the levels of SOD, CAT, and GSH-Px enzymes and lowering MDA levels. ⟡ Prevention of testis cell apoptosis caused by heat stress by blocking the Fas/FasL pathway.	Mouse testis	[140]
Baicalin treatment	⟡ Decreased the expression of ALOX15B, followed by prevention of oxidative stress and apoptosis in porcine Sertoli cells.	Porcine Sertoli cells	[105]
Baicalin treatment (10 µM Baicalein)	⟡ Prevention of tight junction degradation, restoration of mitochondrial function, and reduction in apoptosis caused by heat stress via arachidonic acid in Sertoli cells.	Boar Sertoli cells	[38]
Wuzi Yanzong Pills	⟡ Significant enhancement of Sertoli cell (SC) maturation, viability, and proliferation and improved spermatogenesis. ⟡ Prevention of BTB protein damage and infertility.	Rat Sertoli cells	[192]
Red grape (*Vitis vinifera*) juice (0.8 mL/rat/day)	⟡ Significant enhancement of serum testosterone, testicular SOD, CAT, and testicular glutathione levels. ⟡ Suppressed levels of serum corticosteroid, testicular lipid peroxidase, and the apoptotic enzyme caspase-3 in the testis, along with a substantial decrease in testicular Hsp72 and Hsf-1 and an increase in 17β-HSD3 noted in heat-stressed rats. ⟡ Prevention of germ cell degeneration and tubular deformations, along with restoration of the normal number of sperm, caused by heat stress.	Rat Sertoli cells	[224]
Ginseng (heat-stressed plus KGC04P-200 mg/kg)	⟡ Restored sperm kinetics; facilitated spermatogenesis; reduced the level of inflammatory cytokines; and enhanced the concentration of antioxidant-linked enzymes SOD, CAT, GPX, and GST. ⟡ Prevented testicular damage.	Rat testis	[186,225]
Angelica keiskei (Ashitaba) powder (57.5 mg/kg) and its functional component, xanthoangelol (3 mg/kg)	⟡ Enhanced sperm parameters, including densities of motile sperm, progressive sperm, and amplitude of lateral head displacement. ⟡ Suppressed expression of apoptotic regulatory genes and enhanced the level of the antioxidant enzyme GPX.	Mouse testis	[194]
Saponins derived from the stems and leaves of *Panax ginseng* (150, 300 mg/kg) were administered intragastrically to mice for 14 days	⟡ Reduced the expression of apoptosis-related genes, such as those in the Bcl-2 family and caspase protease family. ⟡ Regulated the MAPK signaling pathway to prevent apoptosis. ⟡ Enhanced antioxidative capacity. ⟡ Improved the process of spermatogenesis in heat-stressed mouse testes.	Mouse testis	[188]
*Platycodon grandiflorum saponin* (PGS) (15, 30 mg/kg) administration intragastrically for 14 days	⟡ Regulated the MAPK signaling pathway to prevent apoptosis. ⟡ Enhanced antioxidative capacity. ⟡ Improved the process of spermatogenesis in heat-stressed mouse testes.	Mouse testis	[190]
Curcumin supplementation (450 and 900 mg/per sheep daily) for 14 days	⟡ Enhanced antioxidant activity (increased GPX levels in plasma). ⟡ Increased testicular weight in Hu sheep. ⟡ Regulated immunity (increasing the concentrations of IgA, IgM, and IgG in plasma). ⟡ Prevented apoptosis by increasing testicular bcl-2 mRNA expression and decreasing caspase-3 mRNA gene expression. ⟡ Elevated the concentration of testosterone in plasma.	Hu sheep testis	[226]
Quercetin and kaempferol	⟡ Protected Sertoli cells from injury caused by heat stress. ⟡ Inhibited the levels of HSP70, ROS, p-NF-κB-p65, and p-IκB in heat-treated Sertoli cells. ⟡ Upregulated the expression of SOD, occludin, vimentin, and F-actin. ⟡ Alleviated heat-induced oxidative stress by enhancing the antioxidant activity of SOD in Sertoli cells.	Sertoli cells	[227]
Betaine (16 mM administration)	⟡ Restored testosterone production, prevented apoptosis (reduced caspase-3 activity), enhanced antioxidant activity (increased the levels of SOD, CAT, GSH-Px), and rescued the reduced serum testosterone concentration in heat-treated mouse Leydig cells.	Mouse Leydig cells	[185]
Tert-butylhydroquinone	⟡ Regulated the expression of NFR2. ⟡ Decreased the levels of malondialdehyde (MDA). ⟡ Enhanced cellular antioxidant ability. ⟡ Reduced oxidative stress. ⟡ Protected against heat-induced testis damage in mice.	Mouse testis	[137]
Tanshinone IIA (TSA)	⟡ Inhibited the expression of TGFβ1/Smad2/Smad3 pathway proteins, preventing cell apoptosis, testicular cell apoptosis, and damage, while enhancing antioxidant activity.	Mouse testis	[117]
Zinc sulfate	⟡ Prevented heat-stress-induced apoptosis and cell injury. ⟡ Improved testosterone synthesis and semen quality.	Ledying cells	[109,228]
Melatonin	⟡ Enhanced the expression of genes associated with apoptosis (SIRT1, SIRT6, and SIRT7).	Human granulosa lutein cells	[229]
Selenium nanoparticle (SeNP) supplementation (0.3, 0.4, and 0.5 mg/kg)	⟡ Enhanced live litter size at birth and weaning, alongside a heightened viability rate at birth. ⟡ Improved hemoglobin levels, red blood cell counts, plasma concentrations of thyroid hormones (T3 and T4), insulin, total proteins, and albumin. ⟡ Enhanced plasma levels of estradiol 17-β, progesterone, and prolactin. ⟡ Improved white blood cell counts, cortisol levels, lipid profiles, and hepatic and renal functions. ⟡ Enhanced immunoglobulin levels, amplified antioxidant capacity, elevated superoxide dismutase levels, and suppressed MDA levels. ⟡ Positively impacted sexual receptivity, pregnancy rates, viability rates at weaning, ovulation rates, and embryo quality.	Rabbit GCs	[80]
Selenium treatment	⟡ Reduced the levels of apoptosis induced by heat stress by suppressing apoptosis-linked genes in mouse granulosa cells. ⟡ The treatment also ameliorated the level of estradiol.	Mouse GCs	[202]
Baicalin treatment	⟡ Decreased MDA content and increased the activities of antioxidant enzymes including SOD, CAT, and GSH-Px of the uterine tissue. ⟡ Regulated Keap1/Nrf2 signaling pathway. ⟡ Nrf2 protein and Nrf2, NQO1, and GCLC mRNA expression levels were significantly increased in the H + Bai group. ⟡ Uterine epithelial cell apoptosis; caspase-3, caspase-9, and Keap1 protein expression levels; and HO-1 mRNA expression levels were decreased in the H + Bai group.	Mouse uterine cells	[98]
Baicalin treatment	⟡ Elevated the ERK1/2 signaling pathway. ⟡ Improved mitochondrial integrity. ⟡ Alleviated apoptosis and oxidative stress. ⟡ Prevented embryo death.	Mouse embryos (blastocyst stage)	[113]
Baicalin treatment	⟡ Reduced the ROS levels and apoptosis and enhanced the mitochondrial membrane potential (ΔΨm) and ATP level. ⟡ Improved the developmental capacity of pig embryos. ⟡ Regulated sonic hedgehog (SHH) signaling which promotes cell proliferation and differentiation in multiple tissues and organs via paracrine signaling.	Pig embryo	[230]
Baicalin treatment	⟡ Significantly increased 2- and 4-cell cleavage rates, morula developmental rate, blastocyst developmental rate and cell number of in vitro-cultured mouse embryos. ⟡ Cell apoptosis was decreased by suppressing the expression of HSP70, CASP3, and BAX.	Mouse embryo	[231]
Baicalin treatment	⟡ Upregulated the expression of Bax and Caspase 3. ⟡ Downregulated the expression of BCL2. ⟡ Suppressed apoptosis of granulosa cells and the ovary. ⟡ Upregulated PI3K/AKT/mTOR signaling.	Mouse GCs and ovary	[232]
Chlorogenic acid	⟡ Improved antioxidant status (inhibited the accumulation of ROS, increased PCNA protein expression and SOD and CAT activities, and reduced MDA level). ⟡ Prevented Sertoli cell apoptosis by downregulating the expression of CASP3 protein and the BAX/BCL-2 protein ratio. ⟡ Mitochondrial membrane potential was enhanced.	Sertoli cells	[116]
Selenium treatment	⟡ Improved the viability of cumulus cells and oocytes. ⟡ Enhanced nfr2 expression which is associated with enhancement of antioxidant response.	Bovine cumulus–oocyte complexes	[90]
Boron	⟡ Blocked heat-stress-induced endoplasmic reticulum stress. ⟡ Suppress the expression of apoptotic-induced genes (caspase-3, GRP78, and CHOP) ⟡ Restored the levels of serum estradiol in vivo.	Mouse granulosa cells	[118]

## 6. Future Research Directions

Future research directions should aim to elucidate the specific mechanisms by which heat-stress-induced oxidative stress and apoptosis impact various mammalian reproductive cells, such as granulosa, Sertoli, and Leydig cells and oocytes. Investigating the molecular pathways responsible for reactive oxygen species (ROS) generation, lipid peroxidation, and apoptotic pathway activation in response to thermal stress is imperative. There is also a pressing need for extensive comparative studies on the effects of heat stress on male and female reproductive cells across diverse mammalian species, including humans, cattle, and other livestock. Unveiling the conserved and distinct cellular responses to heat stress can inform targeted intervention and management approaches.

Additionally, the identification of specific biomarkers indicative of heat stress resilience and diminished apoptosis and oxidative stress is vital. Research should consider the protective roles of molecular chaperones such as heat shock proteins; antioxidative enzymes like SOD and CAT; and signaling pathways including ERK1/2, Nrf2, and AMPK in reproductive cell defense mechanisms. Moreover, evaluating the efficacy of exogenous antioxidants in ameliorating heat-stress-induced cellular damage warrants further investigation, particularly concerning the therapeutic potential of natural and pharmaceutical compounds such as baicalin, anthocyanins, puerarin, and curcumin.

To date, the majority of findings stem from in vitro experiments conducted in controlled settings, which necessitate validation in practical breeding scenarios across mammals. A holistic approach to future studies is essential for developing a comprehensive understanding of heat-stress-induced reproductive cell damage and for devising effective protective strategies to maintain reproductive health in the face of environmental challenges. While current review articles comprehensively elucidate the roles of various antioxidants and biomarkers in conferring heat resistance to mammalian reproductive cells, it is evident that future investigations aiming for a more profound understanding of the molecular mechanisms underpinning these antioxidants and biomarkers are imperative. Such endeavors promise to contribute significantly to our knowledge base and may lead to the development of more effective mitigation strategies against heat-induced stress.

## 7. Conclusions

Heat stress has been demonstrated to have a significant impact on the functionality of mammalian reproductive cells, as supported by existing research. It is a key factor affecting fertility in both humans and animals. Heat stress leads to increased levels of ROS, which disrupt the normal antioxidant defense mechanisms and trigger apoptosis in mammalian reproductive cells. Molecular investigations have revealed that heat stress upregulates the activity of caspase 3 and the pro-apoptotic protein Bax, while simultaneously reducing the expression of the anti-apoptotic protein Bcl-2. Furthermore, this stressor negatively affects signaling pathways such as AMP, NRF2, and ERK1/2, while upregulating MAPK and NF-Kappa B signaling. These molecular alterations contribute to oxidative stress and apoptosis, ultimately suppressing the immune response. Interestingly, supplementation with specific herbal and synthetic antioxidants in the form of medicine or feed additives has shown promise in mitigating the detrimental effects of heat-induced oxidative stress and apoptosis, thereby restoring the normal functioning of reproductive cells. However, it is important to note that while these findings are encouraging, the available data remain in a relatively preliminary stage and require further extensive validation through rigorous research and experimentation.

## Figures and Tables

**Table 1:** Effect of heat stress on mammalian reproductive cells.

Heat Stress	Biological Effect	Cells	Reference
	⟡ Enhanced accumulation of ROS, suppressed SOD, CAT, and proliferating cell nuclear antigen (PCNA) protein expression levels	Sertoli cells	[116]
	⟡ Elevates the level of arachidonic acid which disrupts TJs and enhances oxidative stress and apoptosis ⟡ Disrupts the normal process of spermatogenesis	Sertoli cells	[106]
	⟡ Elevated the transforming growth factor beta 1 (TGFβ1)/SMAD family member (Smad2)/Smad3 pathway protein expression, causing cell apoptosis, testicular tissue organic lesions, and testicular damage and affecting testicular secretion function.	Testis	[117]
	⟡ Increased the process of apoptosis by enhancing the level of Bcl-2-associated X protein (BAX) and Caspase-3 ⟡ Suppressed cell proliferation by downregulating PCNA and CyclinB1 ⟡ Enhanced ROS and oxidative stress by suppressing the level of SOD ⟡ Disrupted the synthesis of progesterone and estrogen by downregulation of the expression of steroidogenic acute regulatory protein (STAR), Cyp11A1	Ovarian granulosa cells	[95]
	⟡ Induced apoptosis via endoplasmic reticulum stress signaling ⟡ Upregulates the expression of apoptotic linked genes (caspase-3, BAX, glucose-regulated protein 78 (GRP78) and CHOP) and downregulates BCL2 gene expression	Mouse granulosa cells	[118]

## Data Availability

All the data are available in the manuscript.

## Abbreviations

HMOX1	Heme oxygenase 1
NOS2	Nitric oxide synthase 2 (inducible)
CAT	Catalase
SOD	Superoxide dismutase
BCL2L1	B-cell lymphoma 2-like 1 (BCL-xL)
GPX4	Glutathione peroxidase 4
Nrf2	Nuclear factor erythroid 2-related factor 2
ASP3	Aspartoacylase (also known as ASPA)
PPARGC1A	Peroxisome proliferator-activated receptor gamma coactivator 1-alpha
SLC16A3	Solute carrier family 16 member 3
SERBP1—SREBP1	Sterol regulatory element-binding protein 1
SIRT1	Sirtuin 1
AMPK	AMP-activated protein kinase
CASP8	Caspase 8
CASP9	Caspase 9
IGF2	Insulin-like growth factor 2
PPARA	Peroxisome proliferator-activated receptor alpha
SLC27A3	Solute carrier family 27 member 3 (also known as FATP3)
NLRP3	NOD-like receptor family pyrin domain-containing 3
STAR	Steroidogenic acute regulatory protein
IRE1	Inositol-requiring enzyme 1
Cyp11A1	Cytochrome P450 family 11 subfamily A member 1
CNA	Calcineurin
CyclinB1	Cyclin B1
Bax	Bcl-2-associated X protein
PCNA	Proliferating cell nuclear antigen
SOD2	Superoxide dismutase 2
ATP5F1A	ATP synthase subunit alpha, mitochondrial
NFE2L2	Nuclear factor erythroid 2-like 2 (also known as Nrf2)
CPT2	Carnitine palmitoyltransferase 2
NQO1	NAD(P)H quinone dehydrogenase 1
TGFβ1	Transforming growth factor beta 1
Smad2	SMAD family member 2
Smad3	SMAD family member 3
